# Mapping the topological organisation of beta oscillations in motor cortex using MEG

**DOI:** 10.1016/j.neuroimage.2018.06.041

**Published:** 2018-11-01

**Authors:** Eleanor L. Barratt, Susan T. Francis, Peter G. Morris, Matthew J. Brookes

**Affiliations:** Sir Peter Mansfield Imaging Centre, School of Physics and Astronomy, University of Nottingham, University Park, Nottingham, NG7 2RD, United Kingdom

**Keywords:** Magnetoencephalography, Post movement beta rebound, Spatial resolution, Beamformer, fMRI, Motor cortex

## Abstract

The spatial topology of the human motor cortex has been well studied, particularly using functional Magnetic Resonance Imaging (fMRI) which allows spatial separation of haemodynamic responses arising from stimulation of different body parts, individual digits and even spatially separate areas of the same digit. However, the spatial organisation of electrophysiological responses, particularly neural oscillations (rhythmic changes in electrical potential across cellular assemblies) has been less well studied. Mapping the spatial signature of neural oscillations is possible using magnetoencephalography (MEG), however spatial differentiation of responses induced by movement of separate digits is a challenge, because the brain regions involved are separated by only a few millimetres. In this paper we first show, in simulation, how to optimise experimental design and beamformer spatial filtering techniques to increase the spatial specificity of MEG derived functional images. Combining this result with experimental data, we then capture the organisation of the post-movement beta band (13–30 Hz) oscillatory response to movement of digits 2 and 5 of the dominant hand, in individual subjects. By comparing these MEG results to ultra-high field (7T) fMRI, we also show significant spatial agreement between beta modulation and the blood oxygenation level dependent (BOLD) response. Our results show that, when using an optimised inverse solution and controlling subject movement (using custom fitted foam padding) the spatial resolution of MEG can be of order 3–5 mm. The method described offers exciting potential to understand better the cortical organisation of oscillations, and to probe such organisation in patient populations where those oscillations are known to be abnormal.

## Highlights

• Aim is to map the topological organisation of neural oscillations in motor cortex.
• MEG spatial resolution optimised by temporal separation of sources.
• Subject motion controlled using foam headcasts.
• Cortical representation of Digit 2 and Digit 5 separated spatially.
• Post movement beta rebound maps motortopically in agreement with BOLD responses.

## Introduction

The topographic organisation of the sensorimotor cortices is well established (Penfield and Boldrey, 1937), and in recent years functional magnetic resonance imaging (fMRI) has allowed a non-invasive means to map the brain's response to stimulation of different body parts (Maillard et al., 2000; Sakai et al., 1995; Stippich et al., 2002), individual digits of the hand (Francis et al., 2000; Gelnar et al., 1998; Sanchez-Panchuelo et al., 2010), and the within digit functional parcellation of Brodmann areas (Sanchez-Panchuelo et al., 2012). This mapping, which separates functionally specialised brain regions lying only millimetres apart, has been made possible by the high spatial resolution of high- and ultra-high field fMRI. However, fMRI can only assess the haemodynamic response, missing the underlying electrophysiology. Non-invasive electrophysiological brain imaging is possible using magnetoencephalography (MEG), which measures the extra-cranial magnetic fields generated by neural current flow (Cohen, 1972). The millisecond temporal resolution of MEG facilitates the capture of time-frequency dynamics, allowing assessment of time and phase locked evoked responses, and time-locked, but non-phase-locked induced changes in neural oscillations (rhythmic fluctuations in electrical potential across neural assemblies). These disparate effects, and their topographical organisation within the sensorimotor cortex, are less well understood. Here, we develop methodology to optimise the spatial resolution of MEG, and use it to investigate the motortopic organisation of neural oscillations.

Successful mapping of cortical organisation requires brain imaging with high spatial precision, however MEG is ‘classically’ thought of as having limited spatial resolution. Firstly, the MEG inverse problem (transforming extracranial magnetic field measurements into images of cortical current) is ill-posed because cortical current at many thousands of voxels must be inferred from measurements at only a few hundred MEG sensors. This introduces an inherent blurring in source space images. Secondly, inaccurate forward modelling can result in the mislocalisation of sources, potentially distorting functional images. Finally, subject movement relative to the MEG sensors results in blurring of the measured fields, and hence reduced spatial precision. These limitations make somatotopic/motortopic mapping a significant challenge for MEG. Nevertheless, there have been successful demonstrations that stimulation of different body parts elicits electrophysiological activity in demonstrably separable cortical regions (Biermann et al., 1998; Brenner et al., 1978; Fisher et al., 2004; Nakamura et al., 1998; Okada et al., 1984). Previous studies have not only shown such separation of the cortical representation of body parts in healthy individuals, but have also demonstrated altered representations following learning (Elbert et al., 1995; Godde et al., 2003; Liu and Ioannides, 2004), peripheral injury (Mogilner et al., 1993; Weiss et al., 2000), and stroke/stroke recovery (Feys et al., 2000; Forss et al., 1999; Gallien et al., 2003; Wikström et al., 2000). Alterations in digit representations have also been shown to be measurable in focal hand dystonia (Elbert et al., 1998; McKenzie et al., 2003) and carpal tunnel syndrome (Druschky et al., 2000; Napadow et al., 2006; Tecchio et al., 2002) showing the clinical potential of such measures. However, to date, most MEG studies focus on mapping the evoked response, with comparatively few studies on neural oscillations.

In sensorimotor cortex, stimulus induced changes in neural oscillations are dominated by the beta band (13–30 Hz) with a robust decrease in power during stimulation (Jasper and Penfield, 1949), followed by a power increase (above baseline) on stimulus cessation (Neuper and Pfurtscheller, 2001). These are known as the event-related beta desynchronisation (ERBD) and post movement beta rebound (PMBR) respectively. The reduction in beta oscillatory power during stimulation has led to theories that high beta power is a marker of neural inhibition. There is good supporting evidence for this, with demonstrable links between beta dynamics and the inhibitory neurotransmitter gamma aminobutyric acid (GABA) (Gaetz et al., 2011; Hall et al., 2011; Muthukumaraswamy et al., 2013). Studies also suggest that neural oscillations play a key role in mediation of communication between brain regions (Fries, 2005; Hunt et al., 2016); indeed a number of well-known resting state networks, including the sensorimotor network, exhibit coordination of beta oscillations across spatially separate nodes (Brookes et al., 2011; Hipp et al., 2012). Further, recent work (Tewarie et al., 2018) suggests that task elicited temporal dynamics of functional connectivity mirror beta power, with the highest long-range connectivity in the sensorimotor system apparent during the PMBR. Collectively, these studies support the hypothesis that the PMBR is an integrative signal (Donner and Siegel, 2011; Liddle et al., 2016) which is a sensitive marker of time resolved connectivity, and which exerts a top down inhibitory influence on the primary sensorimotor regions. The importance of mapping these beta modulations is growing, particularly in light of recent evidence that the PMBR offers the potential to be a biomarker for pathologies, for example schizophrenia (Robson et al., 2016) and multiple sclerosis (Barratt et al., 2017). However, new methodologies are critically required to gain a better understanding of the spatio-temporal signature of beta oscillations, and to realise the significant clinical potential of PMBR measurement.

In this paper, our primary aim is to show (via simulations) that MEG has the required resolution to separate spatially PMBR responses from different digits of the same hand in individual subjects. (Note the requirement to do this in individuals is critical if the clinical potential of these measures is to be realised.) Our secondary aim is to use experimental data to demonstrate that the PMBR exhibits a motortopic organisation in the brain. In what follows we show, both analytically and in simulation, that the spatial resolution of MEG can be improved in cases where experimental design is optimised such that MEG signals, generated at separate cortical locations, can be separated in time, thus facilitating a MEG spatial filtering methodology with enhanced spatial resolution. Secondly, we show experimentally that, if subject motion is controlled (using custom made foam headcasts (Liuzzi et al., 2017; Meyer et al., 2017)), our methodology allows the separation of neural oscillatory responses elicited by movement of two separate digits (the index and little finger) on the same hand. Finally, we compare our MEG results to those obtained using ultra-high-field (7 T) fMRI, to assess the spatial agreement between the MEG derived PMBR and the fMRI derived blood oxygenation level dependent (BOLD) haemodynamic response.

## Simulations: using temporal separation to enhance spatial resolution

### Beamforming

Beamforming (Robinson and Vrba, 1998; Van Veen et al., 1997) is one of the most popular solutions to the MEG inverse problem, particularly for characterisation of the spatial signature of neural oscillations. Using a beamformer, an estimate of current amplitude, $q_{\theta }\left(t\right)$, made at time *t* and a predetermined location and orientation in the brain, **θ**, is given by a weighted sum of MEG sensor measurements such that

[1] $$q_{\theta }\left(t\right)=w_{\theta }^{T}m\left(t\right).$$

Here ***m***(*t*) is a vector of magnetic field measurements made at time *t* across all MEG sensors and **w**_**θ**_ is a vector of weighting parameters tuned to location/orientation **θ**. The superscript *T* indicates a matrix transpose. The weighting parameters are derived based on power minimisation: the overall power in the output signal, $E\left[{(q_{\theta }\left(t\right))}^{2}\right]$, is minimised with the linear constraint that power originating from the location/orientation of interest should remain (note E(x) refers to the expectation value of x). Mathematically the beamformer problem can be written as:

[2] $$min_{w_{\theta }}\left\{E\left[{(q_{\theta }\left(t\right))}^{2}\right]\right\}\;subject\;to\;w_{\theta }^{T}l_{\theta }=1.$$

The source power is given by

[3] $$E\left[{(q_{\theta }\left(t\right))}^{2}\right]=w_{\theta }^{T}Cw_{\theta },$$

where **C** represents the channel level data covariance matrix calculated over a time-frequency window of interest, which must cover all activity of the source to be imaged. **l**_**θ**_ is the forward field vector, which is a vector containing a model of the magnetic fields that would be measured at each of the sensors in response to a source of unit amplitude with location and orientation **θ**. The linear constraint in Equation [2] ($w_{\theta }^{T}l_{\theta }=1$) results directly from this definition of the forward field vector. A solution to Equation [2] is

[4] $$w_{\theta }^{T}=\frac{l_{\theta }^{T}C^{-1}}{l_{\theta }^{T}C^{-1}l_{\theta }}.$$

Sequential application of Equations [3] and [4] to all source locations/orientations across the brain facilitates construction of an image showing the spatial signature of electrical source power. However, a confound is that, with increasing distance from the MEG sensors, noise power tends to dominate genuine source power and this often leads to artefactual inflation of power estimates close to the centre of the brain. For this reason, source power, $E\left[{(q_{\theta }\left(t\right))}^{2}\right]$ is usually normalised by noise power, $E\left[{(n_{\theta }\left(t\right))}^{2}\right]$ power, giving

[5] $$z=\frac{E\left[{(q_{\theta }\left(t\right))}^{2}\right]}{E\left[{(n_{\theta }\left(t\right))}^{2}\right]}=\frac{w_{\theta }^{T}Cw_{\theta }}{w_{\theta }^{T}\Sigma w_{\theta }}$$

where Σ is the estimated sensor level noise covariance matrix which is usually approximated as $\Sigma =\upsilon^{2}I$ (i.e. sensor noise is uncorrelated and has equal amplitude, υ, across all sensors). z is known as the pseudo-z statistic and is employed to give an unbiased estimate of the spatial location of current sources in the brain.

### Separating sources in time and space

**Fig. 1:**
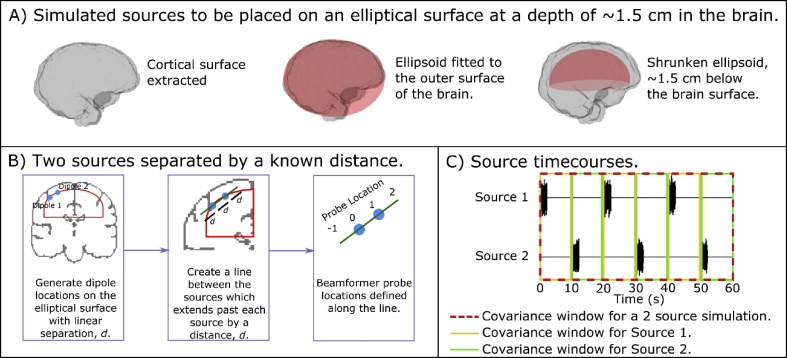
**Schematic of the simulation.** A) The generation of an ellipsoid, approximately 1.5 cm below the brain surface, upon which all sources were simulated. B) Two sources generated in close proximity, and a 1-dimensional beamformer image generated along a line joining the two sources. C) Source timecourses were simulated such that each source was sequentially active, to mimic the experimental set-up used later.

In this paper, we aim to spatially resolve two sources in close proximity. First we examine, in simulation, how the pseudo-z-statistic behaves around two closely spaced sources by modelling two nearby sources located on an ellipsoidal surface inside the brain. Initially, the brain surface was extracted from an anatomical MRI of an individual subject (who had also previously undergone a MEG experiment). An ellipsoid was fitted to the outside surface of the brain, and then shrunk, yielding a second ellipsoid at a depth of approximately 1.5 cm beneath the brain's surface (see Fig. 1A). Simulating all sources on this surface ensured they had equivalent depth in the head, and therefore similar signal to noise ratio (SNR) at the MEG sensors. Two sources were then simulated at random locations on this ellipsoidal surface, separated by a known (linear) distance *d*. Having defined these source locations, we aimed to generate a 1-dimensional beamformer image along a line joining the two sources (see Fig. 1B).

Source timecourses were simulated such that the sources were sequentially active; this was designed to mimic a real experiment in which a paradigm required sequential movement of two digits on the same hand. A single simulated “trial” lasted 20 s; Source 1 was active in the time window 0 < t < 2 s, and Source 2 in the time window 10 < t < 12 s. The timecourses comprised random Gaussian noise multiplied by a boxcar function, such that the strength of source activity was 31 ± 3 nAm (mean ± standard deviation over trials) (see Fig. 1C). A total of 44 trials was used. Each dipole timecourse was multiplied by the forward field, and a noise term added such that the simulated MEG data were given by

[6] $$m_{\mathrm{sim}}\left(t\right)=B_{1}q_{1}\left(t\right)+B_{2}q_{2}\left(t\right)+N\left(t\right)$$

Here, $B_{1}$ is the forward field for Source 1 (with timecourse $q_{1}\left(t\right)$) and $B_{2}$ is the forward field for Source 2 (with timecourse $q_{2}\left(t\right)$). Source orientations were defined to be in a plane tangential to the radial orientation and at an arbitrary angle with respect to the azimuthal orientation. The forward fields were generated using a dipole approximation (Sarvas, 1987) and a multiple local sphere head model (Huang et al., 1999). The position of the head within the MEG helmet was taken from an experimental recording, allowing the necessary co-registration parameters for forward calculations. In all simulations, the sampling rate was 600 Hz. MEG sensor noise was added in two ways:

1) **Gaussian Noise**: Noise was generated as a Gaussian random process (i.e. uncorrelated and equal in strength across all MEG sensors) with 3 amplitudes: 36 fT (low SNR condition); 21 fT (medium SNR condition); 8 fT (high SNR condition).
2) **Empty room Noise**: An 880 s real MEG recording was made, using a 275 channel CTF system, with no subject present, to represent genuine background magnetic interference. To facilitate multiple realisations of these empty room noise data, a multi-variate phase randomisation process was applied (Prichard and Theiler, 1994). This allowed generation of new noise datasets *ad infinitum*, with the same Fourier spectra and linear covariance as the original, whilst scrambling the phases of the Fourier components ensured fundamentally different noise timecourses.

There are multiple ways to employ beamforming to reconstruct these simulated sources, which differ depending on how covariance matrices, and hence beamformer weights, are constructed. Specifically, three possible covariance matrices can be generated: **C**_**1**_ is generated using data in which only Source 1 is active (i.e. data within the 0<t<10s window (for every trial) – yellow boxes in Fig. 1C). **C**_**2**_ is generated using data in which only Source 2 is active (i.e. data within the 10<t<20s window (for every trial) – green boxes in Fig. 1C). **C** is generated using all data (i.e. red box in Fig. 1C). Following this, the beamformer can be constructed in three possible ways.

1) **Single weight vector; Single image**: Only **C** is used to generate the beamformer image: A single set of beamformer weights is generated per source space location/orientation as $w_{\theta }^{T}=\frac{l_{\theta }^{T}C^{-1}}{l_{\theta }^{T}C^{-1}l_{\theta }}$. A single image is then generated as $z_{\theta }=\frac{w_{\theta }^{T}Cw_{\theta }}{{\text{υ}}^{2}w_{\theta }^{T}w_{\theta }}$. This image contains 2 peaks, one for each source.
2) **Single weight vector; Two images**: As above, **C** is used to generate a single set of beamformer weights so that $w_{\theta }^{T}=\frac{l_{\theta }^{T}C^{-1}}{l_{\theta }^{T}C^{-1}l_{\theta }}$. However, two separate images are generated as $z_{1\theta }=\frac{w_{\theta }^{T}C_{1}w_{\theta }}{{\text{υ}}^{2}w_{\theta }^{T}w_{\theta }}$ and $z_{2\theta }=\frac{w_{\theta }^{T}C_{2}w_{\theta }}{{\text{υ}}^{2}w_{\theta }^{T}w_{\theta }}$, which should peak at the spatial location of Source 1 and Source 2 respectively.
3) **Two weight vectors; Two images**: Here, two separate beamformer weight vectors are used to generate the images of the two sources independently. A weight vector, $w_{1\theta }^{T}=\frac{l_{\theta }^{T}C_{1}^{-1}}{l_{\theta }^{T}C_{1}^{-1}l_{\theta }}$, and an image, $z_{1\theta }=\frac{w_{1\theta }^{T}C_{1}w_{1\theta }}{{\text{υ}}^{2}w_{1\theta }^{T}w_{1\theta }}$, are used to spatially localise Source 1, and a second weight vector $w_{2\theta }^{T}=\frac{l_{\theta }^{T}C_{2}^{-1}}{l_{\theta }^{T}C_{2}^{-1}l_{\theta }}$ and a corresponding image $z_{2\theta }=\frac{w_{2\theta }^{T}C_{2}w_{2\theta }}{{\text{υ}}^{2}w_{2\theta }^{T}w_{2\theta }}$, are used to spatially localise Source 2.

Methods 1 and 2 have the advantage that the beamformer weights are reliant on a data covariance matrix which has been constructed using all the available data. This, in principle, makes the covariance matrix more reliable and increases the stability of matrix inversion (required for the weights calculation). However, reducing the number of sources of no interest that a beamformer must minimise facilitates increased spatial resolution. Thus, method 3, in which the weights are derived using covariance matrices which contain data on only a single source, potentially offers an advantage.

**Fig. 2:**
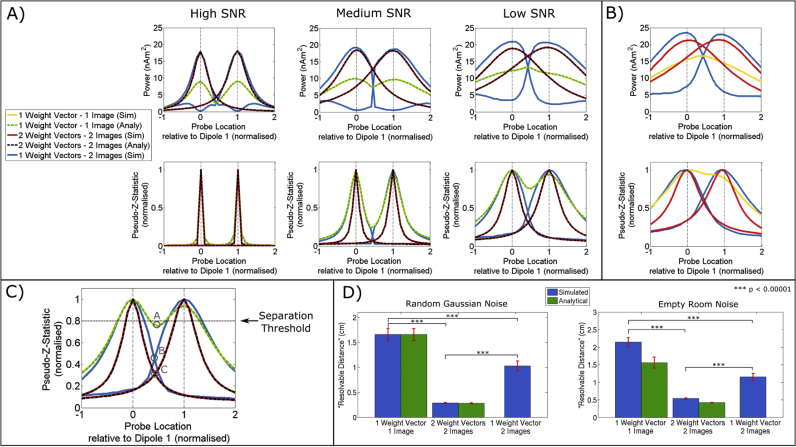
**Simulation results.** A) Beamformer projected power (top) and pseudo-z-image (bottom), for the three beamformer implementations (Single weight vector; single image (yellow). Single weight vector; two images (blue). Two weight vectors; Two images (red) – dashed lines show analytical images computed in the infinite integration limit). The case of high, medium and low SNR are shown in the left, centre, and right plots respectively. Sensor noise is Gaussian. B) Equivalent to (A) but the interference is taken from an empty room. C) Schematic diagram showing how spatial separation of two sources was calculated: the amplitude of the local minima between the peaks (points marked A, B and C) had to be less than 80% of the maximum peak height. D) Spatial resolution for the 3 different beamformer implementations. The left hand plot shows the case for Gaussian noise and the right hand plot shows the case for empty room noise.

Fig. 2A shows beamformer projected power (upper row) and pseudo-z-statistical image (lower row) for Gaussian noise simulations with high (left) medium (centre) and low (right) SNR. The three beamformer methodologies are shown in different colours (Single Weights; Single Image is shown in yellow. Single Weights; Two Images is shown in blue. Two Weights; Two Images is shown in red). In order to compare these simulated methods to a case in which data covariance is perfect (i.e. has no reliance on the amount of data used) we also included analytical computations for methods 1 and 3, shown by the dashed lines (the theoretical equations used to generate these analytical curves are given in Appendix 1). In all cases the beamformer derived pseudo-z-statistical image accurately pinpoints both sources, which here are separated by d = 1.2 cm. However, qualitatively, the sources are better delineated in the case where separate weights vectors are derived based on temporal separation of the sources (method 3). Fig. 2B shows the equivalent plots for empty room noise. Again, qualitatively, deriving separate weights vectors based upon temporal separation of the sources appears advantageous.

To better quantify the results presented in Fig. 2A and B, a more expansive simulation was undertaken. Following random selection of two initial source locations (see Fig. 1B), the distance, d, between the two sources was reduced sequentially. For each value of d, we derived a new beamformer image, with the aim of finding the minimum separation of the two sources (smallest d) when the peaks in the image could be resolved successfully. The condition for resolution is shown schematically in Fig. 2C, and is based upon the local minima between the source locations (i.e. the points labelled A, B, and C in the Figure). For the two sources to be resolved, we required that this local minimum was less than or equal to 80% of the maximum height of the two peaks. The minimum separation was computed independently for the three beamformer implementations. This simulation was repeated 50 times with the sources in different locations on the ellipsoidal surface, and a different noise realisation, on each iteration.

Results are shown in Fig. 2D, for Gaussian Noise (left), and empty room noise (right), with the analytical case also shown, which is in excellent agreement with the Gaussian noise simulation. Note the discrepancy between analytical and simulated results is greater in the empty room noise case since the analytical equations (Appendix 1) cannot mimic the complex (real) sensor-space correlations introduced by real magnetic interference. (I.e. here, to derive the analytical results, we simply matched the noise amplitude level (υ in appendix 1) to the measured standard deviation of the empty room noise, and used the same Equations as for the Gaussian case). Most importantly, for Gaussian and realistic interference, we see a significant decrease in the resolvable distance when splitting the weight vectors in time (Method 3). Quantitatively, the minimum resolvable distanced dropped from 16.5 mm (1 wt vector, 1 image) to 2.8 mm (2 wt vectors, 2 images case) (p < 0.00001 – paired *t*-test) for the Gaussian noise case. For the empty room noise condition, there was again a significant decrease in the resolvable distance from 21.4 mm (1 wt vector, 1 image) to 5.4 mm (2 wt vectors, 2 images case) (p < 0.00001).

These data show that, in cases where paradigm design allows two sources to be separated in time, segmenting the MEG recording into two datasets, to compute separate covariance matrices and beamformer weights, offers better spatial separation. This is a consequence of the minimisation in Equation [2]. When the beamformer is used to probe a specific source location, the derived weights are optimised to minimise other spatially separate sources. However, the fewer sources of no interest there are to minimise, the sharper the peak will be in the beamformer derived image. Let us now suppose that we want to generate an image of Source 1, meaning that Source 2 is effectively an interference source: In methods 1 and 2, beamformer weights are derived based upon all of the data (hence both sources 1 and 2 are represented in the covariance matrix), and the weights are derived to minimise Source 2 (apparent in the blue power curves shown in Fig. 2A). This minimisation works, but increases the full-width at half maximum of the peak over Source 1. However, in method 3, where sources are segmented in time so that weights are based only on a time window in which Source 1 is active, Source 2 is completely invisible and is not represented in the covariance matrix, therefore it doesn't require minimisation. This means that the beamformer is able to better optimise spatial specificity around the source that is active in the time window selected (Source 1), hence improving spatial specificity. Similar arguments have been made previously relating to data averaging (Brookes et al. (2010)). The more sources of no interest that can be eliminated (by segmenting data in time, or in frequency) the better the spatial resolution around sources of interest. However, critically, this is dependent on having sufficient data to construct an accurate data covariance matrix (and its inverse). In cases with fewer trials, these results do not hold (see discussion and supplementary information Figure S2). It should be noted that the quantitative values for spatial resolution presented here are based on dipoles oriented arbitrarily; it has previously been shown (Vrba and Robinson, 2002) that the best spatial resolution between two dipoles will occur when those diploes are oriented perpendicular to one another, whereas parallel dipoles will exhibit the worst resolution. These two cases represent the extrema; our simulation (using arbitrary angles) represents an average.

## The topographical organisation of beta oscillations: experimental method

Using insights from the simulations above, we aim to use a MEG beamformer to determine the topological organisation of the PMBR in motor cortex: Specifically, to show, in individual subjects, significant spatial separation of responses to moving either the second digit (D2 – index finger), or the fifth digit (D5 – little finger) of the dominant (right) hand.

### Participants

Three healthy adults (1 female – age 35 years; 2 male – aged 26 and 24 years) took part in the study, having given full written consent. The study was approved by the University of Nottingham Medical School Research Ethics Committee. Each subject was scanned 8 times in the MEG system across 2–4 days. The same subjects took part in a separate fMRI session to allow comparison between the spatial topology of beta band neural oscillations and the BOLD response.

### Paradigm design

**Fig. 3:**
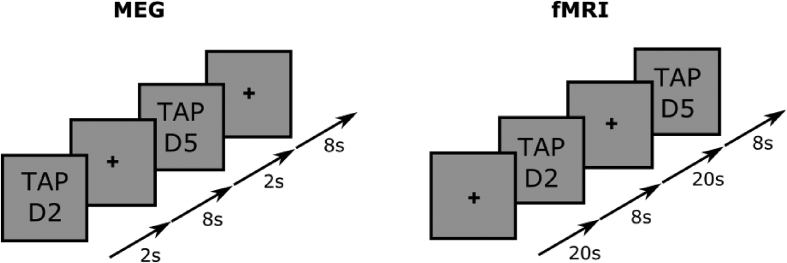
Paradigm design for the digit tapping experiment used in MEG (left) and fMRI (right).

Finger movement paradigms were optimised for MEG and fMRI measurements separately. In both cases, a visual cue instructed subjects to “tap” (execute flexion and extension) either D2 or D5 against a surface at a frequency of approximately 2–4 Hz (though this was not monitored explicitly). For MEG, the paradigm was designed to mimic the simulations and thus optimise spatial resolution. Each trial lasted 20 s and comprised 2 s of tapping D2, followed by 8 s rest, and then 2 s of tapping D5, followed by a further 8 s rest (see Fig. 3, left hand side), with 44 trials collected. The entire MEG experiment was then repeated 8 times in each subject. For fMRI, subjects were again instructed to tap D2 or D5, but given the latency and longevity of the haemodynamic response and poor temporal resolution of fMRI, tapping of each digit was performed for 8 s, followed by a 20 s rest period between digits (see Fig. 3, right hand side) with 6 trials collected. In order to enhance SNR sufficiently to allow digit separation in fMRI, where necessary, this experiment was repeated, and data concatenated across repeats. Subject 1 took part in one scan (6 trials); Subject 2 took part in 4 scans (24 trials); and Subject 3 took part in 2 scans (12 trials). A high-resolution T_2_^∗^- weighted scan was acquired following fMRI to allow detection of the veins.

### Data acquisition: MEG

The representations of D2 and D5 in motor cortex are separated spatially by only a few millimetres. Given this, it was important to limit subject movement within the MEG helmet throughout the ∼15-min recording which could obscure measurement of digit separation. Thus, we constructed subject-specific foam headcasts, the internal surface of which fitted closely to the subjects' scalp, whilst the external surface fitted to the MEG scanner helmet. Similar headcasts have been shown experimentally to reduce subject motion to ∼1 mm (Liuzzi et al., 2017; Meyer et al., 2017).

To generate the headcast, an anatomical MRI of the subject's head was acquired using a 3T Philips Ingenia scanner running a 3DTFE sequence with resolution of 1 mm^3^. Sequence parameters (TR/TE = 4.5 ms/1.97 ms; FOV (AP, FH, RL) = 256 × 256 × 183 mm; BW = 775 Hz) were optimised to provide a high bandwidth/voxel and hence minimise spatial distortion due to susceptibility effects around the face and scalp. A 3D mesh representing the outer surface of the head and face was extracted from the anatomical MRI. This 3D model of the subject's head was placed inside a virtual realisation of the MEG helmet. The head-model was augmented with spacing elements, to optimise the distance between the scalp and sensors. In addition, a 3D representation of three head localisation coils, used for localising head position in the MEG scanner was added, with coils placed at the nasion and left and right preauricular points. This 3D digital representation of the head surface, spacers, and localisation coils was 3D printed and placed inside a manufacturer-provided replica of the MEG-helmet. Finally, liquid resin was poured into the negative space between the surfaces. This expands and sets, resulting in flexible foam headcasts which can also house the localising coils.

All MEG data were acquired using a 275-channel whole head CTF system (MISL, Coquitlam, Canada) operating in third order gradiometer configuration at a sampling frequency of 600 Hz. The subject was seated throughout the recording. Prior to acquisition, three localisation coils were placed in the headcast. These were energised continuously during the scan, allowing tracking of any movement of the headcast relative to the scanner. The headcast design involved a complete digital representation of the subject's brain anatomy, relative to the localisation coils. Further, energising these coils allowed knowledge of the locations of the three coils relative to the MEG sensor locations. Combining the headcast geometry with the MEG data thus allowed co-registration between the system geometry and the brain anatomy. Visual cues for the MEG paradigm were presented via projection through a waveguide onto a back-projection screen placed ∼40 cm in front of the subject.

### Data acquisition: fMRI

All fMRI data were acquired using a Philips 7T Achieva system (Philips Medical Systems) with a head only volume transmit coil and 32-channel receive coil (Nova Medical). Foam padding was placed around each subject's head to minimise movement. Data were acquired using a gradient-echo, echo-planar-imaging (GE-EPI) sequence with the following acquisition parameters: TR = 2 s, echo time (TE) = 25 ms, flip angle (FA) = 75° (Ernst angle), SENSE factor 2.5. 26 axial slices, with 1.5 mm^3^ isotropic resolution (FOV = 183×39×183 mm^3^) were acquired to cover the sensorimotor regions. Throughout the scan, visual cues for the fMRI paradigm were presented via projection onto a back-projection screen placed at the end of the magnet bore which subjects viewed via a pair of prism glasses.

### Data analysis: MEG

All MEG data were inspected visually and any trials containing excessive interference (e.g. due to the magnetomyogram) were removed – this resulted in the reduction of the trial count to 42 ± 2 in Subject 1; 41 ± 3 in Subject 2 and 42 ± 2 in Subject 3 (mean and standard deviation across 8 runs in each subject). The mean (across 8 runs) maximum movement for subjects 1, 2 and 3 was 0.3 mm, 1.2 mm and 0.3 mm respectively, illustrating the distinct advantage of using a headcast; no trials were removed due to subject movement. Data were frequency filtered between 1 and 150 Hz.

Following preprocessing, MEG data were modelled using a scalar beamformer, as described above. Forward fields were computed using a dipole approximation (Sarvas, 1987) and a multiple-local-spheres head model (Huang et al., 1999). Data were projected into source space onto the vertices of a regular 2 mm grid spanning the entire brain – facilitating a volumetric image. Given the results of our simulations, in order to optimise spatial specificity, separate beamformer weights were calculated to represent each digit independently: weights for D2 ($w_{D2})$ were constructed using data covariance measured in the 0<t<10s window (relative to the start of all trials). Weights for D5 ($w_{D5})$ were generated using data covariance measured in the 10<t<20s time window (again relative to the start of all trials). In both cases, data were frequency filtered to the beta (13–30 Hz) band. Since the number of effective samples (degrees of freedom) in the data is given by $2B_{W}\text{Δ}$, where $B_{W}$ is the signal bandwidth and Δ is the total duration of data (in seconds), this means a total of (2×17(Hz)×10(s)×44(trials)=) 14,960 samples with which to quantify covariance; this is considered sufficient according to a previous theoretical study (Brookes et al., 2008).

The spatial signature of beta modulation was determined using a pseudo t-statistical approach, which contrasts ‘active’ and ‘control’ windows. The image for D2 was generated using

[7] $$T_{\mathrm{\theta D}2}=\frac{w_{D2}^{T}C_{\mathrm{aD}2}w_{D2}-w_{D2}^{T}C_{\mathrm{cD}2}w_{D2}}{2w_{D2}^{T}w_{D2}}\text{.}$$

Since we aimed to image the spatial distribution of the PMBR, the active window to generate $C_{\mathrm{aD}2}$ was taken from 2.5 to 4 s and the control window to generate $C_{\mathrm{cD}2}$ from 7.5 to 9 s. Similarly, pseudo t-statistical images showing the spatial distribution of PMBR for D5 tapping were given by

[8] $$T_{\mathrm{\theta D}5}=\frac{w_{D5}^{T}C_{\mathrm{aD}5}w_{D5}-w_{D5}^{T}C_{\mathrm{cD}5}w_{D5}}{2w_{D5}^{T}w_{D5}}\text{,}$$

where the active window to generate $C_{\mathrm{aD}5}$ was 12.5–14 s and the control window to generate $C_{\mathrm{cD}5}\;$ was 17.5–19 s. The main focus here is on the PMBR, but see Figure S1 for the spatial localisation of the ERBD. In Equations [7] and [8], the denominator was constructed to remove bias towards the centre of the brain.

These analyses produced pseudo-*t*-statistical images from which to determine a peak location (location of the largest response) of the PMBR, in left sensorimotor cortex, for each of the 8 experimental runs in each participant independently. The coordinates of these peaks were determined in each subject's ‘native’ MEG space. Given the known motortopic organisation of the motor cortex we expected that the representation of D5 would appear superior to the representation for D2. Thus, a one-sided paired *t*-test was applied to the z-co-ordinate (foot to head) to determine if the peak locations were significantly different spatially, following tapping of D2 compared to D5. In keeping with our aim to show motortopic organisation in individuals, this test was performed on each subject separately as well as at the group level. For visualisation purposes, these peak locations were overlaid onto coronal, saggittal, and axial slices of the individual subjects MRI.

### fMRI

Data were motion corrected using MCFLIRT (Jenkinson et al., 2002) and high-pass filtered at 0.01 Hz. No spatial smoothing was applied to the 7T fMRI data to minimise loss of spatial resolution. fMRI data were analysed using a GLM (FSL FEAT (Jenkinson et al., 2012):) with the motion parameters used as covariates of no interest in the design matrix, and an additional variable included to ensure no confound of concatenation of data across multiple runs in the same subject. Contrasts of D2 and D5 activation, as well as a differential contrast was performed between the digits (D2>D5) and (D5>D2) giving z-score maps for each contrast. These z-score maps were then combined by masking the differential contrasts with the individual functional images for D2 and D5. In addition, a venous mask was created from T2*-weighted images, which was applied to the z-score maps to remove any voxels comprising draining vein effects. The resulting z-score maps were then transformed linearly into the MEG data space, allowing direct comparison of the spatial signature of BOLD changes to the MEG pseudo-*t*-statistical maps. All differential contrasts were originally thresholded to a z-score of 1.96. For visualisation purposes, these were then further masked by the activation maps at a level dependent upon the noise of each subject and digit (for Subject 1, both digit masks were thresholded at z = 2.3; for Subject 2, the D2 mask was thresholded at z = 2.3, while the D5 mask was thresholded at z = 5.2); and for Subject 3, D2 was thresholded at z = 2.3 while D5 was thresholded at z = 5.2).

### Comparison of MEG and fMRI spatial maps

The MEG and fMRI results were compared quantitatively by measuring the distance between peak locations of digits. Specifically, for each subject, we measured the distance between each MEG-D2 peak from each experimental run, and the corresponding D2 and D5 fMRI peaks. We hypothesised that MEG-D2-to-fMRI-D2 distance should be smaller than the MEG-D2-to-fMRI-D5 distance. This numerical analysis was repeated for D5, and data were combined. These analyses yielded a “*corresponding digit distance*" (i.e. MEG-D2-to-fMRI-D2 and MEG-D5-to-fMRI-D5) and an “*alternate digit distance*" (MEG-D2-to-fMRI-D5 and MEG-D5-to-fMRI-D2). These values were assessed to determine if the corresponding digit distance was smaller than the alternate digit distance, using a one sided paired *t*-test. Again, in keeping with our aim to achieve spatial separation of MEG responses in individuals, this metric was measured for each individual subject as well as at the group level.

## The topographical organisation of beta oscillations: results

**Fig. 4:**
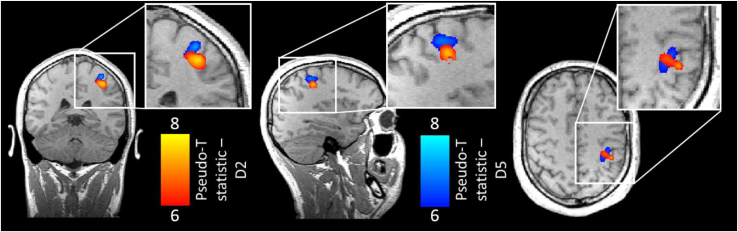
Example pseudo-T-statistical images, from a single run in a single subject. The spatial distribution of the PMBR measured following D2 movement is shown in red. The PMBR following D5 movement is shown in blue. Note, qualitatively, a spatial shift in the two responses with the representation of D5 appearing superior, as expected from the known organisation of the sensorimotor cortex.

**Fig. 5:**
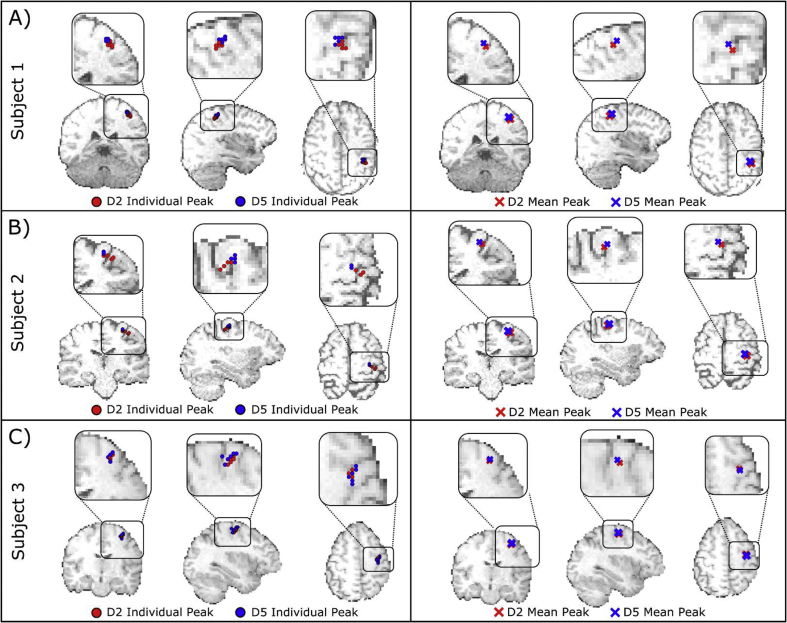
**MEG experimental results:** The spatial distribution of the PMBR response, shown for Subjects 1–3 in panels A–C respectively. In all cases, the spatial distributions of PMBR responses for D2 tapping are shown in red, for D5 tapping in blue. In the left hand panel, peak locations for all 8 runs are shown in each subject, projected onto axial, coronal and sagittal slices of the MRI. In the right hand panel, the average locations (across all 8 runs) are shown.

Fig. 4 shows example pseudo-T-statistical images for a representative run in a single subject. The spatial representations of PMBR following D2 and D5 tapping are shown in red and blue respectively. Note, as expected, a spatial shift is apparent with the representation of D5 superior to that of D2. The peak PMBR locations from the pseudo-T-statistical maps for all runs and all subjects are shown in Fig. 5; PMBR peaks following D2 tapping are shown as red circles, and PMBR peaks following D5 tapping are shown as blue circles (Fig. 5, left panel). These peak locations have been projected onto single axial, coronal and saggittal slices for visualisation. The pseudo-T statistical values (mean ± standard deviation) for D2 tapping were 9.5 ± 2.5, 10.3 ± 1.8 and 11.0 ± 2.3 for subjects 1–3 respectively. The equivalent pseudo-T statistical values for D5 tapping were 11.9 ± 2.3, 10.0 ± 1.4 and 9.7 ± 2.6. The right hand panel of Fig. 5 shows the mean PMBR peak locations (across runs) for D2 and D5 as red and blue crosses respectively.

For all subjects, there was a significant superior shift in peak location of D5 (blue) compared to D2 (red) as expected from the known organisation of motor cortex. For Subject 1, the average superior-inferior (S-I) separation between D2 and D5 peaks was 3.5 ± 0.7 mm (p = 0.001); for Subject 2, the S-I separation was 2 ± 1 mm (p = 0.03); and for Subject 3, the S-I separation was 1.5 ± 0.3 mm (p = 0.001). (The Euclidean separation of the peaks was 5.5 ± 1 mm for Subject 1, 4.3 ± 2 mm for Subject 2 and 2.8 ± 0.4 mm for Subject 3). Treating the three subjects as a group (i.e. performing a 1 tailed *t*-test over the 24 individual measurements) resulted in a significant S-I shift with p = 0.00001. At the group level there was also a significant (p = 0.01) left-right shift in peak locations, with D5 peak appearing more medial (as expected), however this medial shift did not reach statistical significance in individual subjects. Importantly, these significant separations of the representation of D2 and D5 were only observable when deriving optimised beamformer weights, capturing D2 and D5 movement separately. If a single weights vector was employed, the spatial shifts were diminished significantly (see Appendix 2 and Discussion).

**Fig. 6:**
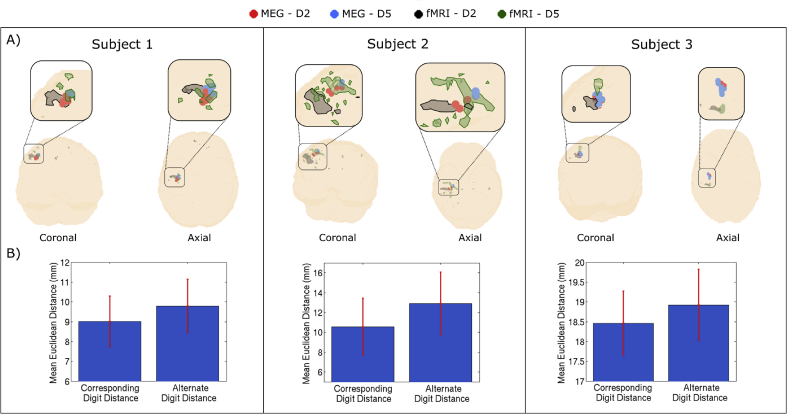
**The spatial relationship between the PMBR and the BOLD response.** A) The spatial location of the 8 MEG derived peak locations of the PMBR are shown by red (D2) and blue (D5) circles. The grey and green overlays show the peak BOLD signal change in response to D2 and D5 respectively. All differential contrasts were thresholded at a z-score of 1.96, before further thresholding using the activation maps. B) The bar charts show the corresponding digit distance (i.e. MEG-D2-to-fMRI-D2 and MEG-D5-to-fMRI-D5) and alternate digit distances (i.e. MEG-D2-to-fMRI-D5 and MEG-D5-to-fMRI-D2); note as expected that the corresponding digit distance is smaller than the alternate digit distance in all subjects (although this is only significant in Subjects 1 and 2).

Fig. 6 shows the spatial relationship between the MEG derived PMBR and the BOLD response. In Fig. 6A, the red and blue circles show PMBR peaks for D2 and D5 respectively, for all 8 experimental runs in each subject. The grey overlay shows the BOLD response elicited by D2 tapping; the green overlay shows the BOLD response elicited by D5 tapping. Coronal and axial projections are shown. Note that, whilst there is clear discrepancy (of approximately 1 cm) between the PMBR and BOLD locations, particularly in Subject 3, the superior shift between D2 and D5 is apparent in both modalities. The locations of the MEG and fMRI derived peaks, in MNI space, are given in Table 1. Fig. 6B shows the corresponding and alternate digit distances. As expected, the corresponding digit distance was lower than the alternate digit distance, although this was only significant in Subjects 1 and 2. In Subject 3, there was a larger discrepancy, with the largest BOLD response being located in sensory rather than motor cortex. At the group level, the mean corresponding digit distance was 12.6 mm compared to a mean alternate digit distance of 13.9 mm; the significant (p = 0.003, one-sided paired *t*-test) difference between these values shows that, on average, the PMBR has a spatial organisation which is similar to that of the BOLD response.

**Table 1:** **MNI coordinates and locations of the peak PMBR and BOLD responses.** In the case of PMBR, the mean across all 8 runs is given alongside the SD in brackets. The locations are based on MNI indexing.

		MNI x-coordinate	MNI y-coordinate	MNI z-coordinate	Location
Subject 1	PMBR – D2	−37 (1)	−24 (2)	51 (1)	Post central gyrus
	PMBR – D5	−35 (1)	−20 (2)	54 (1)	Pre central gyrus
	fMRI – D2	−40	−20	50	Post central gyrus
	fMRI – D5	−36	−18	68	Pre central gyrus
Subject 2	PMBR – D2	−35 (5)	−15 (4)	53 (2)	Pre central gyrus
	PMBR – D5	−32 (1)	−13 (1)	56 (1)	Pre central gyrus
	fMRI – D2	−50	−22	42	Post central gyrus
	fMRI – D5	−36	−14	52	Pre central gyrus
Subject 3	PMBR – D2	−32 (2)	−11 (3)	58 (2)	Pre central gyrus
	PMBR – D5	−31 (2)	−11 (4)	60 (3)	Pre central gyrus
	fMRI – D2	−38	−28	54	Post central gyrus
	fMRI – D5	−32	−28	68	Post central gyrus

## Discussion

A number of studies have demonstrated the ability to spatially differentiate cortical responses to movement or stimulation of a given digit using fMRI (Besle et al., 2014; Kolasinski et al., 2016; Martuzzi et al., 2014; Sanchez-Panchuelo et al., 2010; Stringer et al., 2011). In contrast, the spatial resolution of MEG has traditionally been thought of as limited due to the ill-posed nature of the inverse problem, inaccuracies in modelling magnetic fields generated by electrical sources, and practical problems such as subject movement, making motortopic mapping of the digits challenging. In this paper, via optimisation of the spatial specificity of a beamformer, and by exploiting the use of custom made foam head padding to reduce subject movement, we were able to spatially separate the PMBR response induced by the movement of two separate digits (the index (D2) and little (D5) finger) of the same hand, in individual subjects. Specifically, our results show a statistically significant superior shift in the spatial location of the PMBR response to D5 relative to D2 movement across 8 independent MEG experiments, as would be expected given the known spatial organisation of the motor cortex. This was repeated in three individuals. Further, we showed significant agreement between the PMBR and the measured location of the BOLD fMRI response.

The beamformer methodology was key to the spatial separation of the PMBR of D2 and D5. The beamformer method places minima over sources that are not generated at the spatial location being probed (the minimisation term in Equation [2]). This ability to adaptively remove sources of no interest is one of the significant advantages of this approach (Sekihara et al., 2006). However, the more sources of no interest it must minimise, the lower the spatial specificity around a true source will be. It is therefore a significant advantage to segment data, in either time or frequency, to remove sources of no interest prior to construction of the beamformer weights vectors. This was the approach taken here; the data were segmented temporally into periods when only a single source was active, and the beamformer weights were tuned specifically to that source. This resulted in a significant improvement in spatial resolution in our simulations, and was critical in showing robust experimental separation of responses to D2 and D5 movement. In the absence of such segmentation (i.e. when a single beamformer weights vector is defined using data covariance calculated across the whole experiment) our simulations showed a drop in spatial resolution. Moreover, in our experimental data, separation of the cortical representations of D2 and D5 was no longer possible in two out of the three subjects, and Euclidean separation was reduced significantly across all subjects (See appendix 2).

Exploitation of this segmentation approach comes with the caveat that sufficient data must have been collected to allow temporal (or spectral) separation. Using fewer data points, the accuracy of the covariance matrix will degrade, which will cause the beamformer projected power to deviate from its expected value. This is illustrated in Figure S2 in which beamformer reconstructed power, summed across a 1-dimensional image, is measured in simulation (i.e. using data derived covariance) and analytically (where covariance has no dependence on the volume of data used). Any deviation of the data-derived versus analytical projected power will degrade the spatial accuracy of the beamformer reconstruction. It can be seen that, as the number of trials, and hence amount of data, is reduced, for methodologies in which data are segmented, the power deviates quicker from the expected result than for cases where the data are not segmented (Brookes et al., 2008). Thus, to exploit fully a temporal segmentation of sources to optimise spatial specificity, sufficient data are required for each source independently; this should be taken into consideration in the experimental design for future studies.

Arguably the greatest limitation of spatial resolution in MEG is subject movement relative to the MEG sensors, causing blurring of the measured fields and hence a reduction in the accuracy of the inverse models that are used to reconstruct the data. In typical studies, motion tolerance is often set at 5 mm (challenging for many subjects over a 15 min MEG recording). However, the spatial separation of D2 and D5 sources was expected to be of this order, requiring headcasts to limit subject movement. The headcasts limited head motion to ∼1 mm throughout the measurement, sufficient to allow digit separation in motor cortex. These flexible foam pads are an attractive option to reduce head motion at source. However, it should be noted that they are expensive, and the complete elimination of the ability to move the head may make the scanner experience intolerable for some subjects, in particular patients. For this reason, practical use of MEG to measure motortopic organisation in patient populations might require other solutions. Recent years have seen the development of methods for post-hoc correction of MEG data recorded in the presence of head movement (Medvedovsky et al., 2007; Nenonen et al. 2010, 2012; Taulu and Simola, 2006; Uutela et al., 2001). However, these methods cannot deal with the inherent change in SNR to sources brought about by movement relative to sensors (i.e. a large left to right shift would give left hemisphere sources decreased SNR and right hemisphere sources increased SNR). In addition, motion correction of the data tends to reduce degrees of freedom which itself might lower the spatial resolution of the beamformer. This therefore poses a fundamental problem for the current generation of MEG. However, the introduction of novel field sensors and head mounted arrays which can be worn whilst subject moves naturally (Boto et al., 2016; Boto et al., 2017; Boto et al., 2018), offers a practical solution.

Our data showed agreement (at the group level and in two of the three subjects scanned) between the spatial representation of the PMBR and the BOLD response. This finding adds weight to a body of work which suggests that oscillatory changes might, at least in part, drive the metabolic demand which elicits haemodynamic changes (Brookes et al., 2005; Hall et al., 2014; Logothetis et al., 2001; Winterer et al., 2007). However, there was not a perfect overlap between the MEG findings and the BOLD response. This could relate to a bias in the forward model used for MEG - we have assumed a point dipole model for the underlying sources, however it is clear that, in practice the PMBR will be generated from a finite volume of tissue. This potentially means that the precise locations of the MEG sources may undergo a systematic shift because the dipole model cannot capture the complex geometry of activation in an extended volume of cortex. This may also explain why the spatial separation of D2 and D5 responses was greater in fMRI (15 ± 3 mm) than in MEG (4 ± 1 mm). This said, the discrepancy between PMBR and BOLD could also be neurophysiological in origin. The BOLD response represents integrated cortical activity, which in the case of our paradigm will exist in both the sensory and the motor regions. However, the PMBR may relate more directly to motor control; indeed there is some evidence for this since spatially, the PMBR has robustly been shown to originate from a location anterior in the brain to the commonly measured ERBD, with the former originating predominantly from the precentral gyrus, and the latter ostensibly from the post central sulcus (Barratt et al., 2017; Fry et al., 2016; Jurkiewicz et al., 2006; Stancák Jr and Pfurtscheller, 1995). It is conceivable that, if the PMBR represents motor activity, a spatial shift between this and the BOLD response (which shows both motor and sensory activation) wouldn't be surprising. Indeed, this is the case in Subject 3 where the PMBR occurs in primary motor cortex (pre-central-sulcus) and the peak BOLD response is localised to primary sensory cortex (post-central-sulcus). Future studies, in which electrophysiological forward models are based on an activated volume rather than a point dipole will be valuable in determining the precise origins of these spatial shifts. Indeed, the use of fMRI to derive a volumar activation model would likely be of significant interest.

Finally, we should recognise the clinical potential of these metrics. We chose to focus on the PMBR specifically due to recent findings of its perturbation in disease. For example, Barratt et al. previously showed the timing of the PMBR to be delayed significantly in patients suffering from multiple sclerosis (Barratt et al., 2017), whilst Robson et al. showed the PMBR to be significantly reduced in amplitude in patients with schizophrenia (Robson et al., 2016). Furthermore, Hunt et al. (Hunt et al., In Press) have shown that the magnitude of the PMBR changes with personality type, being reduced in those subjects who showed high schizotypy (a personality variant which subjectively relates experiences and behaviour to that of schizophrenia patients). All of these studies probed the time-frequency dynamics of the PMBR, but not its spatial organisation across digits or body parts. The ability to spatially separate PMBR responses in the cortex may therefore be important in these disorders. Moreover, there are a number of disorders associated with abnormal hand movement, such as focal hand dystonia and carpel tunnel syndrome, where understanding the spatial organisation of the cortex could be valuable clinically. The demonstration and methods presented here may therefore help in the management of these patients.

## Conclusion

We have shown that by using an optimised beamformer and custom made headcasts to limit subject movement, MEG can offer sufficient spatial specificity to probe the motortopic organisation of the human brain. We showed that the PMBR elicited by little finger movement mapped significantly superior in the cortex to that elicited by index finger movement. Comparison of these results to ultra-high field fMRI shows the two modalities to be in significant agreement. Critically, the spatial separation of cortical digit representations was only obtainable if beamformer weights vectors were derived independently for each source. This means that in future studies aiming to maximise spatial resolution of MEG beamforming, researchers should incorporate careful experimental design in order to temporally (or even spectrally) segment sources, and thus take advantage of this effect. From a neuroscientific perspective, these results shed further light on the nature of the PMBR, and offer exciting potential to understand the topological cortical organisation of neural oscillations in healthy individuals, and in patient populations where the PMBR is known to be altered.

## Figures and Tables

**Fig. A1:**
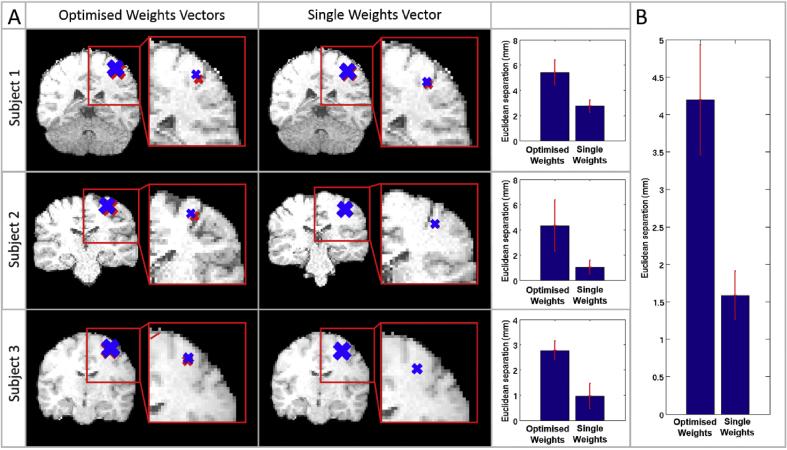
A) Left hand column shows the mean location of the peak PMBR response for D2 (red) and D5 (blue) in the optimised covariance case. The centre column shows the mean location of the peak PMBR response for D2 (red) and D5 (blue) using a generic weights vector. The right hand column shows Euclidean separation of D2 and D5 cortical representations in both cases. Separate rows show three different subjects. B) Euclidean separation between D2 and D5, averaged across subjects, for the two different weights calculations.

## Appendix 1 The theoretical distribution of beamformer projected source power

Consider a single source with a forward field $B_{1}$ and r.m.s. amplitude, $q_{1}$. The analytical form of the data covariance matrix is given by

[A1] $$C_{1}=\;\upsilon^{2}I+q_{1}^{2}B_{1}B_{1}^{T},$$

where the first term represents sensor level noise (which we assume to be uncorrelated and equal across sensors; $\upsilon^{2}$represents noise power, and I denotes the identity matrix). The second term represents the genuine source covariance. The Sherman-Morrison-Woodbury matrix inversion lemma (Sherman and Morrison, 1950; Woodbury, 1950) allows inversion of this matrix, giving

[A2] $$C_{1}^{-1}=\frac{1}{\upsilon^{2}}\left[I-\;\frac{q_{1}^{2}B_{1}B_{1}^{T}}{\upsilon^{2}+q_{1}^{2}{\left|\left|B_{1}\right|\right|}_{F}^{2}}\right]$$

The beamformer estimated source power, at some ‘probe’ location and orientation **θ** is given by

[A3] $$P=w_{\theta }^{T}Cw_{\theta }={\left[l_{\theta }^{T}C_{1}^{-1}l_{\theta }\right]}^{-1}$$

Substitution of Equation [A2] into Equation [A3] (see Brookes et al., 2008) gives

[A4] $$P=\;\frac{\;\upsilon^{2}}{{\left|\left|l_{\theta }\right|\right|}_{F}^{2}\left(1-Gcos^{2}\lambda \right)}$$

where $cos\lambda =\;\frac{B_{1}^{T}l_{\theta }}{{\left|\left|B_{1}\right|\right|}_{F}{\left|\left|l_{\theta }\right|\right|}_{F}}$ is effectively a measure of the linear correlation between the modelled lead field at the probe location $l_{\theta }$ and the forward field for the source $B_{1}$. $G=\;\frac{q_{1}^{2}{\left|\left|B_{1}\right|\right|}_{F}^{2}}{\upsilon^{2}+q_{1}^{2}{\left|\left|B_{1}\right|\right|}_{F}^{2}}$. Equation [A4] shows, analytically, the behaviour of beamformer reconstructed source power at any location around a single source.

It is possible to expand this single source case to two sources. Here, we assume two dipolar sources with forward fields $B_{1}$ and $B_{2}$ and source amplitudes $q_{1}$ and $q_{2}$ respectively. Assuming uncorrelated noise across sensors, the source covariance is given by

[A5] $$C_{12}=\;\upsilon^{2}I+q_{1}^{2}B_{1}B_{1}^{T}\;+\;q_{2}^{2}B_{2}B_{2}^{T}\;,$$

and the inverse covariance is (Brookes et al., 2009)

[A6] $$C_{12}^{-1}=\;\frac{1}{\upsilon^{2}}\;\left[I-\;\frac{1}{1-F_{1}F_{2}\;\cos^{2}\left(\lambda_{12}\right)}\;\left(F_{1}\frac{B_{1}B_{1}^{T}}{{\left|\left|B_{1}\right|\right|}_{F}^{2}}+\;F_{2}\frac{B_{2}B_{2}^{T}}{{\left|\left|B_{2}\right|\right|}_{F}^{2}}-F_{1}F_{2}\;\cos\left(\;\lambda_{12}\right)\;\;\frac{B_{2}B_{1}^{T}+B_{1}B_{2}^{T}}{{\left|\left|B_{1}\right|\right|}_{F}{\left|\left|B_{2}\right|\right|}_{F}}\right)\right]$$

where

[A7] $$F_{1}=\;\frac{q_{1}^{2}{\left|\left|B_{1}\right|\right|}_{F}^{2}}{\upsilon^{2}+q_{1}^{2}{\left|\left|B_{1}\right|\right|}_{F}^{2}}$$

[A8] $$F_{2}=\;\frac{q_{2}^{2}{\left|\left|B_{2}\right|\right|}_{F}^{2}}{\upsilon^{2}+q_{2}^{2}{\left|\left|B_{2}\right|\right|}_{F}^{2}}$$

and

[A9] $$\cos\left(\lambda_{12}\right)=\;\;\frac{B_{1}^{T}B_{2}}{{\left|\left|B_{1}\right|\right|}_{F}{\left|\left|B_{2}\right|\right|}_{F}}$$

Substituting Equation [A6] into the expression for beamformer projected source power yields an expression for source power in the 2 source case

[A10] $$P=\;\frac{\nu^{2}}{{\left|\left|l_{\theta }\right|\right|}_{F}^{2}}\left[\frac{1-F_{1}F_{2}\;\cos^{2}\left(\lambda_{12}\right)}{1-{\text{F}}_{1}\;\cos^{2}\left(\alpha \right)-F_{2}\;\cos^{2}\left(\beta \right)-F_{1}F_{2}\text{cos}\left(\lambda_{12}\right)\left\{\text{cos}(\lambda_{12}-2\;\cos\left(\alpha \right)\text{cos}\left(\beta \right)\right\}}\right]$$

where

[A11] $$\cos\left(\alpha \right)=\;\frac{B_{1}^{T}l}{{\left|\left|B_{1}\right|\right|}_{F}{\left|\left|l_{\theta }\right|\right|}_{F}}\text{,}$$

and

[A12] $$\cos\left(\beta \right)=\;\frac{B_{2}^{T}l}{{\left|\left|B_{2}\right|\right|}_{F}{\left|\left|l_{\theta }\right|\right|}_{F}}\text{.}$$

Here, the cos(α) term is related to the correlation between sensor level spatial topographies of Source 1 and the forward solution at the probe location. Similarly, cos(β) is related to the sensor level correlation between Source 2 and the probe location.

## Appendix 2 Spatial characterisation of PMBR using non-optimised covariance calculation

Results in Fig. 4, Fig. 5, Fig. 6 show that, using an optimised covariance calculation (with separate beamformer weights defined for D2 and D5 movement) we were able to spatially separate the representation of the index and little finger of a subject's dominant hand. In this appendix we show that, without our optimised weights strategy, this spatial separation is more challenging.

Our MEG data for all subjects were reanalysed using the same beamformer spatial filtering approach with sensor space data projected into source space onto the vertices of a regular 2 mm grid spanning the entire brain. However, rather than separate beamformer weights for each experimental condition (i.e. $w_{D2}$ constructed using covariance measured in the 0<t<10s window and $\;w_{D5}$ generated using covariance measured in the 10<t<20s time window), here we constructed a generic set of weights, $\;w_{Gen}$, using data from the whole trial. The spatial signature of beta modulation was then determined using the same pseudo t-statistical approach, contrasting ‘active’ and ‘control’ windows. The image for D2 was generated using

[A13] $$T_{\mathrm{\theta D}2}=\frac{w_{Gen}^{T}C_{\mathrm{aD}2}w_{Gen}-w_{Gen}^{T}C_{\mathrm{cD}2}w_{Gen}}{2w_{Gen}^{T}w_{Gen}}\text{.}$$

As previously, $C_{\mathrm{aD}2}$ was taken from the 2.5–4 s window and $C_{\mathrm{cD}2}$ was taken from the 7.5–9 s window (timings with respect to trial onset). Similarly, pseudo t-statistical images showing the spatial distribution of PMBR for D5 tapping were given by

[A14] $$T_{\mathrm{\theta D}5}=\frac{w_{Gen}^{T}C_{\mathrm{aD}5}w_{Gen}-w_{Gen}^{T}C_{\mathrm{cD}5}w_{Gen}}{2w_{Gen}^{T}w_{Gen}},$$

where $C_{\mathrm{aD}5}$ was generated in the 12.5–14 s window and $C_{\mathrm{cD}5}\;$was generated in the 17.5–19 s. Notice that Equations [A13], [A14] are identical to Equations [7], [8]), apart from the fact that a single beamformer weights vector, $w_{Gen}$, was employed to generate both images (i.e. this is equivalent to the “single weights vector – two images” case in our simulations).

In Figure A1A, the left hand column shows the mean (across all 8 experimental runs) location of the peak PMBR response for D2 (red) and D5 (blue) in the optimised covariance case, with images generated using Equations [7], [8]) (this is equivalent to the result shown in Fig. 5). The centre column shows the same thing, but with a generic weights vector defined based upon covariance calculated across the whole experiment. The right hand column shows Euclidean separation of the D2 and D5 cortical representations in both cases. The three rows in the Figure show the case for three separate subjects. Figure A1B shows Euclidean separation between D2 and D5 cortical representations, averaged across subjects. We observed a significant (p = 0.0019) decrease in spatial separation when using a single weights vector, compared to the optimised weights. Further, the significant superior shift in representation of D5 compared to D2, that we had observed in our primary results in all three subjects, only remained significant in a single subject (1) when using a single weights vector. This result therefore shows the critical importance of the optimised covariance strategy if the full potential for high spatial resolution mapping in beamformer imaging is to be realised.

## Appendix A Supplementary data

SupplementaryInformation SupplementaryInformation
