# Supplementary material for: Mapping the topological organisation of beta oscillations in motor cortex using MEG
Source: Neuroimage. 2018 Nov 1;181:831–44. doi: 10.1016/j.neuroimage.2018.06.041 (PMC6150950; doi:10.1016/j.neuroimage.2018.06.041)
Supplement: SupplementaryInformation [file mmc1.pdf]

Supplementary Information

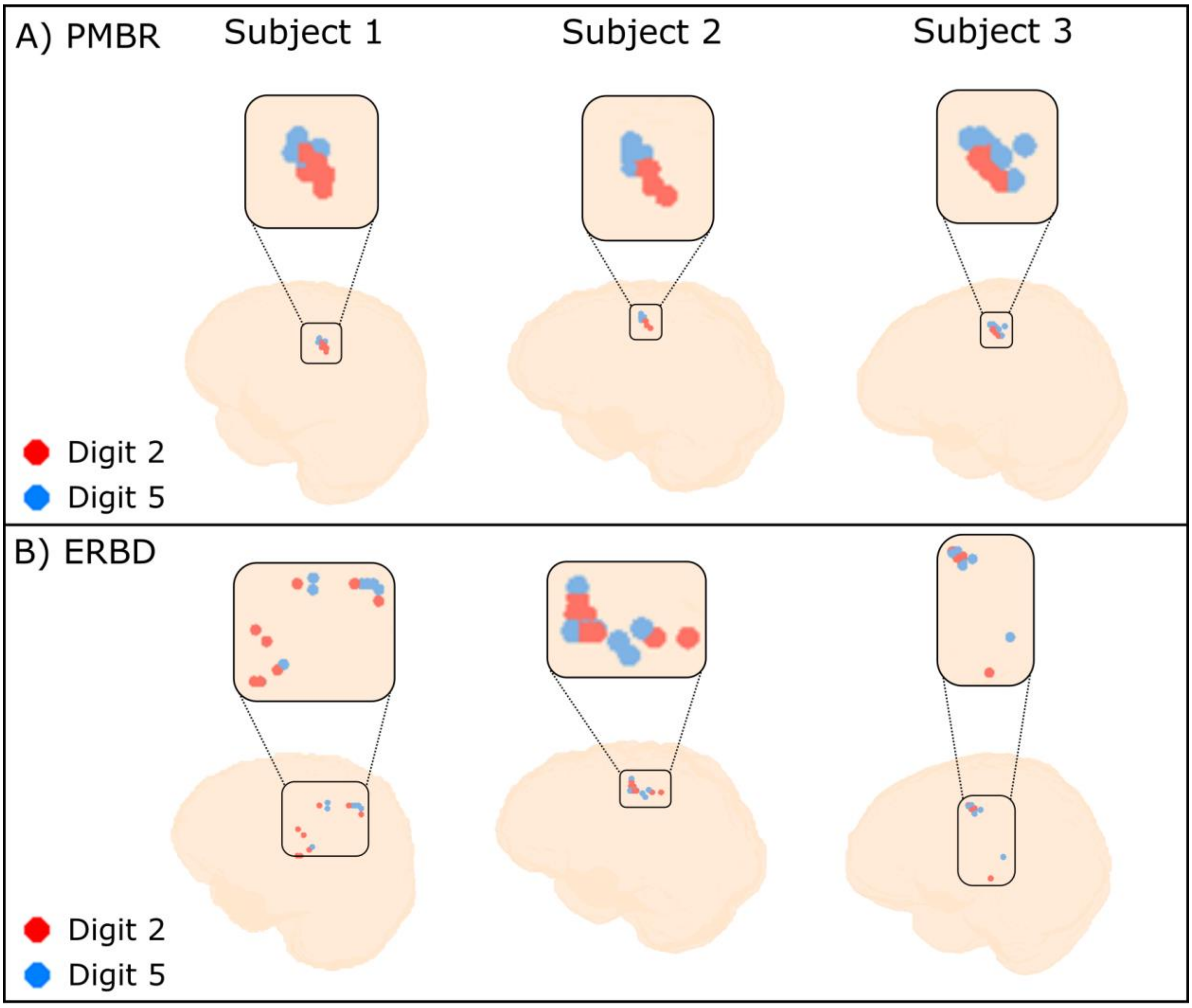

Figure S1: Spatial locations of the p*eak MEG pseudo t-statistical images of the PMBR (A) and ERBD (B) for the 3 subjects.* *Digit 2 peak locations are shown in red, and Digit 5 peak locations are shown in blue. Insets shows a zoomed-in area around the region of interest. To image the PMBR for Digit 2 we used an active window of 2.5-4 s, and a control window of 7.5-9 s relative to trial onset. To image the PMBR for Digit 5 we used an active window of 12.5-14 s, and a control window of 17.5-19 s relative to trial onset. For the ERBD, for Digit 2 we used an active window of 0-2 s, and a control window of 7-9 s, and for Digit 5 we used an active window of 10-12 s, and a control window of 17-19 s. Separate beamformer weights were calculated to represent each digit independently: Weights for D2 were constructed using data covariance measured in the 0 s<t<10 s window (relative to the start of the trial). Weights for D5 were generated using data covariance measured in the 10 s<t<20 s time window. In all cases, data were frequency filtered to the beta (13-30 Hz) band. Although a clear pattern is seen in the digit locations for the PMBR, all ordering appears to be lost for the ERBD.*

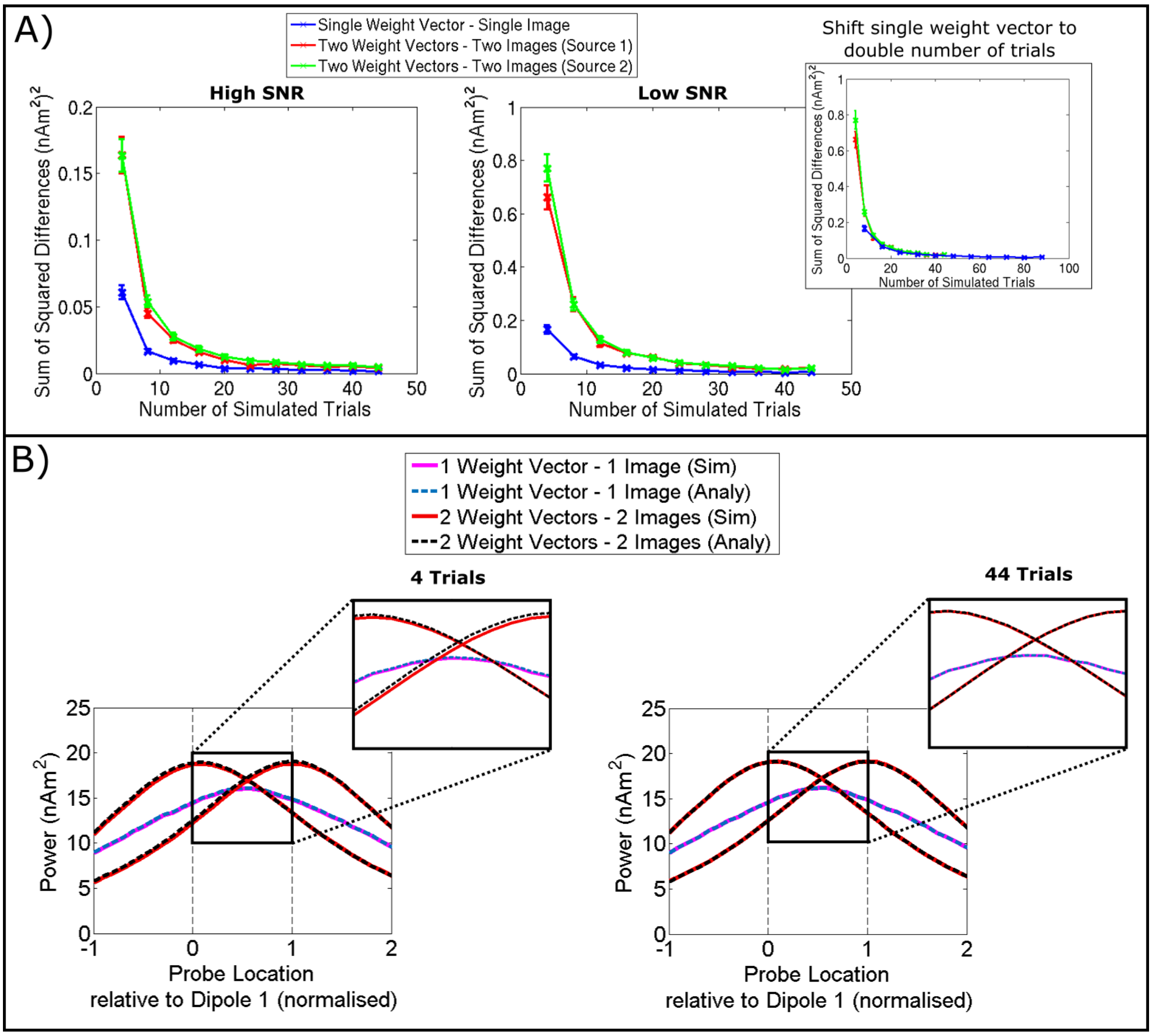

Figure S2: Effect of simulated trials on power. *A) The sum of the squared differences between simulated and analytical power as a function of the number of simulated trials of data. Two dipoles were placed on the surface of the ellipsoid located between 1-1.5 cm apart, and simulations were run as described in our main manuscript, but with the number of trials varying from 4 to 44 for each location. This was run for 50 iterations for both the high (left) and low (right) SNR conditions. The squared difference between the simulated and analytical power were summed over all probe locations. A greater sum of squared differences is seen when less trials are simulated, particularly for the two weight vector cases (red and green), where the data volume is half that of the single weight vector case (blue). This is highlighted by the inset figure for the low SNR case, where the single weight condition (blue line) has been shifted to double the amount of trials, and now overlaps the single weight conditions. Error bars show SE across iterations. B) Example power plots for simulated data with 4 trials (left) and 44 trials (right). When the number of trials is reduced, a clear difference is seen between the simulated (solid line) and analytical (dashed line) power, which is not apparent for the 44 trial case. Again, the difference is greater for the two weight vectors cases compared with the single weight vector case.*

.
